# Effects of whole-body vibration on sensorimotor deficits and brain plasticity among people with chronic ankle instability: a study protocol for a single-blind randomized controlled trial

**DOI:** 10.1186/s13102-023-00698-0

**Published:** 2023-07-18

**Authors:** Jingwang Tan, Jiatao Li, Jianbin Lei, Shuyi Lu, Yongjian Feng, Tao Ma, Lijiang Luan, Roger Adams, Yagang Song, Jia Han, Yu Zou

**Affiliations:** 1 Department of Sport and Exercise Science, College of Education, Zhejiang University, 886 Yuhangtang Road, Zhejiang, 310058 Hangzhou China; 2 School of Elite Sport, Shanghai University of Sport, Shanghai, China; 3 School of Exercise and Health, Shanghai University of Sport, Shanghai, China; 4 Research Institute for Sport and Exercise, University of Canberra, Canberra, Australia; 5 Department of Physical Education Teaching, Shanghai Sanda University, Shanghai, China; 6 College of Rehabilitation Sciences, Shanghai University of Medicine and Health Sciences, 279 Zhouzhu Highway, Shanghai, 201318 China; 7 Faculty of Health, Arts and Design, Swinburne University of Technology, VIC Hawthorn, Australia

**Keywords:** Whole-body vibration, Chronic ankle instability, Sensorimotor deficits, Brain plasticity, Study protocol, Randomized controlled trial

## Abstract

**Background:**

Chronic ankle instability (CAI) is a form of musculoskeletal disease that can occur after a lateral ankle sprain, and it is characterized by pain, recurrent ankle sprains, a feeling of “giving way” at the ankle joint, and sensorimotor deficits. There has been increasing evidence to suggest that plastic changes in the brain after the initial injury play an important role in CAI. As one modality to treat CAI, whole-body vibration (WBV) has been found to be beneficial for treating the sensorimotor deficits accompanying CAI, but whether these benefits are associated with brain plasticity remains unknown. Therefore, the current study aims to investigate the effect of WBV on sensorimotor deficits and determine its correlation with plastic changes in the brain.

**Methods:**

The present study is a single-blind randomized controlled trial. A total of 80 participants with CAI recruited from the university and local communities will be divided into 4 groups: whole-body vibration and balance training (WBVBT), balance training (BT), whole-body vibration (WBV), and control group. Participants will be given the WBV intervention (25-38 Hz, 1.3-2 mm, 3-time per week, 6-week) supervised by a professional therapist. Primary outcome measures are sensorimotor function including strength, balance, proprioception and functional performance. Brain plasticity will be evaluated by corticomotor excitability, inhibition, and representation of muscles, as measured by transcranial magnetic stimulation. Activation of brain areas will be assessed through functional near-infrared spectroscopy. Secondary outcome measures are self-reported functional outcomes involving the Cumberland Ankle Instability Tool and the Foot and Ankle Ability Measure. All tests will be conducted before and after the WBV intervention, and at 2-week follow-up. Per‑protocol and intention-to-treat analysis will be applied if any participants withdraw.

**Discussion:**

This is the first trial to investigate the role of brain plasticity in sensorimotor changes brought by WBV for individuals with CAI. As plastic changes in the brain have been an increasingly important aspect in CAI, the results of the current study can provide insight into the treatment of CAI from the perspective of brain plasticity.

**Trial registration:**

Chinese Clinical Trial Registry (ChiCTR2300068972); registered on 02 March 2023.

## Background

About 20–50% of people with a history of lateral ankle sprain may develop chronic ankle instability (CAI) [1, 2], which is characterized by pain, the feeling of "giving way" at the ankle joint, and recurrent ankle sprains [3]. In addition, CAI is accompanied by sensorimotor deficits [4], shown by reduced balance, strength, and muscle activity. The long-term sequelae of CAI are problematic, as it can lead to financial burdens [5], limited physical activity [6], and reduced health-related quality of life [7] for patients.

Despite the growing body of research evidence, the underlying mechanism of CAI is still unclear [3, 8, 9]. It was previously suggested that the sensory dysfunction that occurred after the initial lateral ankle sprain would affect sensorimotor function of patients with CAI [4]. However, the understanding of CAI has gradually evolved from a structural-pathology paradigm focusing on peripheral structural changes to a neurophysiological paradigm concentrating on the neuronal processing of noxious stimuli [10, 11]. According to theories proposed by Needle [9] and Heterl [3], it was speculated that peripheral sensory deficits such as inflammation, pain, instability, and laxity would lead to subsequent sensorimotor deficits through causing functional and structural changes in the brain. For instance, individuals with CAI were found to possess reduced corticomotor excitability in a transcranial magnetic stimulation test [12], different cortical activation evaluated by functional near-infrared spectroscopy [13], and an altered brain network revealed in resting-state functional magnetic resonance imaging [14]. Additionally, structural evidence has also been observed including microstructural damage in white matter [15], reduced grey matter volume [16] and impairment in the corticospinal tract [17]. The above alterations are important because they are closely related to the changes of sensorimotor deficits for patients with CAI [18–20]. Fortunately, these plastic changes can be restored through specific interventions. A recent study found that stroboscopic balance training could not only improve postural control, but also modulate cortical activities of CAI athletes [21]. Likewise, another study reported that transcutaneous electrical nerve stimulation was effective in modifying corticospinal excitability in individuals with CAI [22]. Accordingly, plastic changes in the brain should be considered when treating CAI symptoms.

Neuromuscular training, which usually refers to either proprioceptive drill, strength training, or a combination of both [23, 24], is an effective intervention modality in treating the sensorimotor deficits of individuals with CAI [25, 26]. However, it has not been fully determined as to which neurophysiological mechanism contributes the most to the benefits of neuromuscular training. Based on the existing hypothesis, the mechanism underlying the effect of neuromuscular training on sensorimotor deficits may be related to both spinal (e.g., altered H-reflex) and supraspinal adaptation (e.g., feed-forward reflexive mechanism) [4, 27, 28], yet relevant evidence is lacking for these hypotheses. As one form of neuromuscular training, whole-body vibration (WBV) has been widely studied in different areas such as sports [29], rehabilitation [30], and public health [31]. The possible mechanism of WBV benefits is that it could facilitate reflexive activation at both the spinal and supraspinal level in the central nervous system [32, 33]. To be specific, WBV has been shown to be capable of modulating spinal excitability in a human trial [34], and modulating synaptic plasticity [35, 36] and neurogenesis [37] in the brain of animals. Regarding the effectiveness of WBV for CAI, it has been found that additional benefits could be achieved in sensorimotor deficits (e.g., postural control, strength, and muscle activity) when WBV was added as a supplementary tool [38–40]. Nevertheless, it was still unknown whether the improvements in sensorimotor functions were associated with plastic changes in the brain for people with CAI. Investigation of the relationship between effectiveness of WBV and brain plasticity may provide understanding regarding the pathways through which WBV works on CAI [16].

Therefore, a single-blind randomized controlled trial will be designed to answer the following questions: (a) if WBV can generate sensorimotor benefits for patients with CAI? (b) is there a correlation between changes of sensorimotor functions and alterations of brain plasticity for participants with CAI? In this study, We hypothesize that there will be plastic changes in the brain after the WBV intervention, and these alterations will be correlated with changes in sensorimotor functions.

## Methods

### Study design and ethical approval

The present study is a single-blind randomized controlled trial designed following the.

**Fig. 1:**
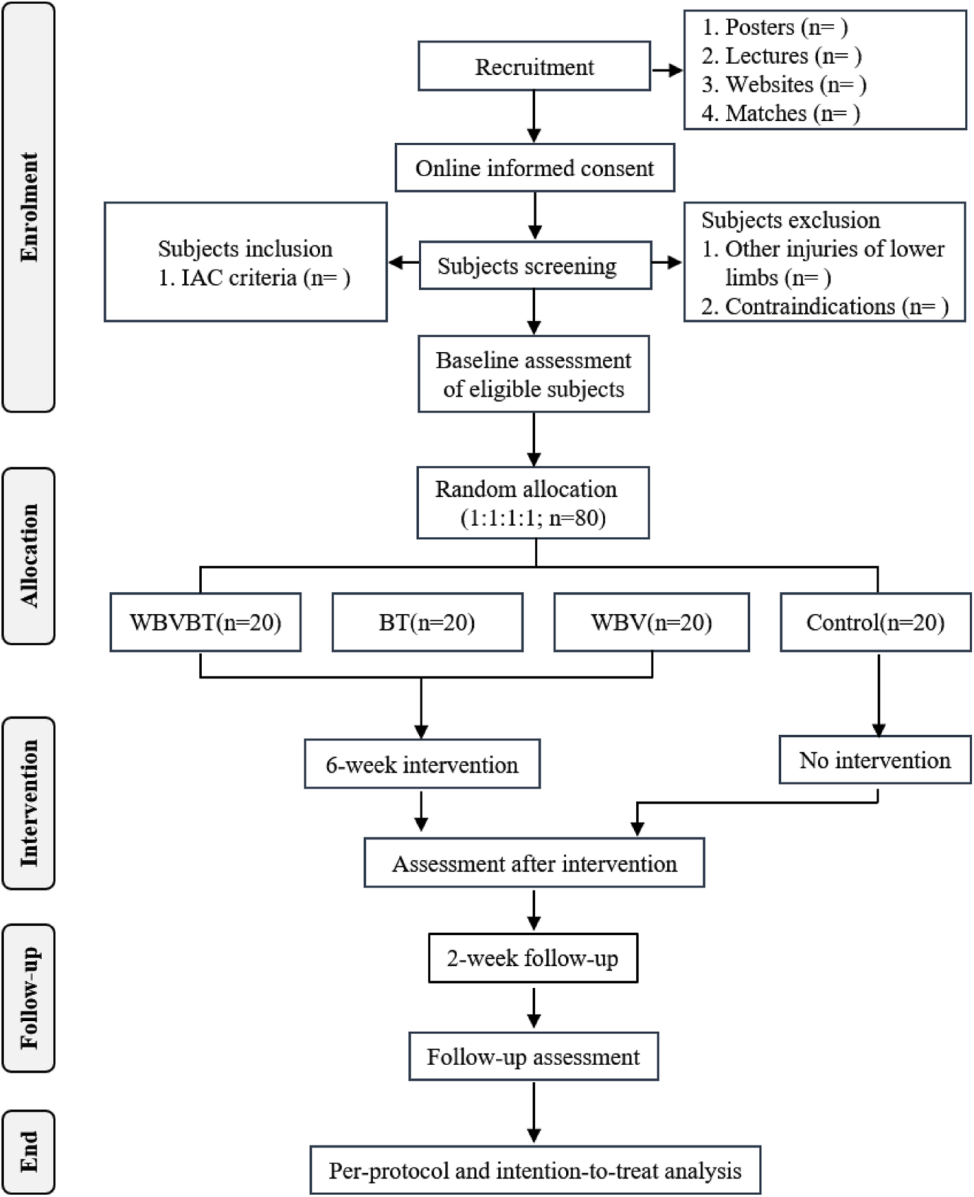
Flowchart showing participant recruitment, interventions, and assessments. Abbreviations: BT = Balance Training; IAC = International Ankle Consortium; WBV = Whole-Body Vibration; WBVBT = Whole-Body Vibration and Balance Training

Consolidated Standards of Reporting Trials (CONSORT, version 2010) [41], Standard Protocol Items Recommendations for Interventional Trials (SPIRIT, version 2013 [42, 43] and PRO Extension [44, 45]) (See supplementary file 1). The flowchart, schedule of enrollment, intervention and assessments are presented in Fig. 1 and Table 2 respectively. The research protocol has been approved by the Medical Ethics Committee of the Department of Psychology and Behavioral Science of Zhejiang University (NO. 2,022,095). In addition, the current study was registered at the Chinese Clinical Trial Registry (ChiCTR2300068972) on 02 March 2023.

### Participants

Participants will be recruited from universities and nearby communities. The selection criteria used in the recruitment procedure will be in accordance with the position statement of the International Ankle Consortium [46]. The inclusion criteria for the CAI group will be: (1) a history of at least one significant ankle sprain with the first sprain occurring at least 12 months before the study; (2) the sprain is associated with inflammatory symptoms (e.g., pain, swelling, etc.) and interrupts desired physical activities for at least 1 day; (3) participants report that there are at least two episodes of the ankle joint “giving way” and/or recurrent ankle sprains in the 6-month period before the study; (4) feeling of instability confirmed by the Cumberland Ankle Instability Tool (CAIT < 24), and the score < 90% in the section of activities of daily living and < 80% in the section of sports activities of the Foot and Ankle Ability Measure (FAAM). The exclusion criteria for the CAI group will be: (1) pregnancy; (2) vestibular or cognitive deficits; (3) neurological (e.g., concussion) or musculoskeletal (e.g., anterior cruciate ligament reconstruction) problems that probably affecting ankle movements or balance; (4) contraindications to participate in assessments such as transcranial magnetic stimulation (e.g., microprocessor implant, epilepsy).

### Randomization, allocation, and blinding

Concealed random allocation will be conducted by an independent researcher after the baseline testing using a sealed envelope filled with random numbers (1–99) generated by the software of Microsoft Excel.

Eligible participants will be allocated into 4 groups (Fig. 1):

1. balance training (BT): undertaking balance training on the flat floor.
2. whole-body vibration (WBV): standing on the vibration platform using one-legged stance and conduct the same tasks as BT group.
3. whole-body vibration and balance training (WBVBT): performing balance training on the vibration platform.
4. control group: no intervention.

Participants in all groups will be allowed to maintain their normal physical activity but avoid any therapeutic exercises of the ankle joint. As WBV stimulation can be easily felt, it is difficult to blind patients. Accordingly, blinding will only be performed on assessors.

### Intervention

The WBV intervention programs of this trial are structured according to previously published guidelines [47, 48] and will be conducted with the supervision of a professional therapist in the fitness center of Zhejiang University. In this study, the vibration stimulus will be generated in synchronous form by one platform with a handrail (Myo 2, Power Plate Corp., USA) and another one without a handrail (A6, TiFit Corp., China). Before the experiment, the participants will be instructed to perform a warm-up program consisting of 5-min of stretching and 5-min of bicycling. Verbal and visual instructions will be given to the participants before performing any sort of exercise. The limb with the lower CAIT score will be chosen if the individuals have bilateral CAI. The angle of the knee in the WBV group will be set at 20-30º to reduce the transmission of vibrations to the head as much as possible. The frequency (number of cycles per second) is adjustable and will be calibrated each time prior to the experiment using an accelerometer (66B, VICTOR Corp., China) placed at the central point of the platform surface. Table 1 indicates the intervention scheme used in the present study.

**Table 1:** Six-week whole-body vibration intervention program for people with chronic ankle instability

Week	Exercise	Duration-set- rest period	Magnitude; Hertz
1	One-legged stance on the foam surface	60 s-5–60 s	1.3 mm; 25 Hz
2	One-legged stance on the BOSU	60 s-5–60 s	1.3 mm; 25 Hz
3	One-legged stance on the foam surface and holding a medicine ball head of the cheast (1 kg)	60 s-5–60 s	2 mm; 30 Hz
4	One-legged stance on the foam surface and throwing a medicine ball upwards (1 kg)	60 s-5–60 s	2 mm; 30 Hz
5	One-legged stance on the BOSU and holding a medicine ball head of the cheast (1 kg)	60 s-5–60 s	2 mm; 38 Hz
6	One-legged stance on the BOSU and throwing a medicine ball upwards (1 kg)	60 s-5–60 s	2 mm; 38 Hz

According to principles of neural plasticity, components including intensity, task-specificity, focus of attention, repetition, and duration will be modified to achieve the desired neuroplastic alterations [49, 50]. As a previous study found that a vibration frequency at 27 Hz rather than 10 Hz generated greater activation in the cerebral cortex [51], the vibration frequency spectrum will range from 25 to 38 Hz, with the magnitude (peak-to-peak displacement) of 1.3-2 mm to achieve the intensity of vibration stimulation. Balance training, in which participants stand on the foam or BOSU placed on the vibration platform, will be added to WBV intervention as a way of obtaining an increased degree of intensity. To meet the principle of task-specificity, participants in all experimental groups will be requested to stand with involved leg. External focus will be given by asking subjects to hold a medicine ball ahead of the chest as steady as possible and catch a medicine ball thrown upwards (5–10 cm of height) by the participants themselves. As for the repetition, the training program conducted 3-day a week will consist of 5 series of 60-s exercises with 60-s rest period between on each day [52, 53]. Previous study has found that acute WBV does not seem able to yield significant changes for individuals with CAI [54, 55], so a long-term training program lasting for 6-week will accordingly be scheduled, based on reference to previous studies [21, 39].

### Outcome measures

Sociodemographic information including name, sex, age, dominant leg, physical activity and level of kinesiophobia will be collected through online questionnaires. Self-reported measures consist of the CAIT and FAAM. Objective measures in the current study include sensorimotor function and brain plasticity. The primary outcome measures will be balance with SEBT end-point to determine sensorimotor deficit, and activation of brain area with oxyhemoglobin in determining brain plasticity. All measures of outcome will be performed by a qualified blinded assessor, who is independent of the current study, at the fitness laboratory of Zhejiang University. An adequate rest period, assessed with a rating of perceived exertion (Borg scale, 6–20), will be given between each of the two objective measures to avoid potential fatigue. Since TMS is potentially associated with alterations of functional performance, the test orders (Table 2) will be arranged to mitigate any mutual influence between each two tests. To avoid the influence of myopia on balance control, participants will be asked to wear any corrective eyewear throughout the test process. The extremity with the lower CAIT score will be tested if bilateral ankle instability exists. Outcome measures will be assessed before and after the WBV intervention. According to a previous study, there were delayed improvements in balance and self-reported functions for patients with CAIafter accepting balance training programs [56]. Hence, the follow-up assessments will be performed 2-week after the intervention program.

**Table 2:** SPIRIT timetable including schedule of enrolment, interventions and assessments

	Study period					
	Enrolment	Baseline assessment	Allocation	Post-allocation	Post-intervention assessment	Follow-up
Time points	-4 to -1 weeks		0	1–6 Weeks	6–7 weeks	8–9 Weeks
Enrollment						
Eligibility screen	X					
Informed consent	X					
Random allocation			X			
Intervention						
WBV						
Assessment						
Primary outcome measures						
Brain activity						
Corticomotor Plasticity (TMS)		X			X	X
Activation of brain area (fNIRS)		X			X	X
Sensorimotor deficits						
Balance (SEBT&TIBT)		X			X	X
Strength (HHD)		X			X	X
Proprioception (AMEDA)		X			X	X
Functional performance (SHT)		X			X	X
Secondary outcome measures						
Ankle instability (CAIT)		X			X	X
Ankle function (FAAM)		X			X	X

Abbreviations: *AMEDA* Active Movement Extent Discrimination Apparatus, *CAI* Chronic Ankle Instability, *CAIT* Cumberland Ankle Instability Tool, *FAAM* Foot and Ankle Ability Measure, *HHD* Hand-Held Dynamometer, *fNIRS* Functional Near-Infrared Spectroscopy, *SEBT* Star Excursion Balance Test, *SHT* Side-Hop Test, *SPIRIT* Standard Protocol Items Recommendations for Interventional Trials, *TIBT* Time-In-Balance Test, *TMS* Transcranial Magnetic Stimulation, *WBV* Whole-Body Vibration

### Sensorimotor deficits

Static balance will be estimated through the Time-In-Balance Test (TIBT). In the TIBT, participants will stand on a firm surface in a single-legged stance. Participants will be instructed to put their hands on their spina iliaca. The time taken to keep balance with eyes closed will be recorded and referenced with a cutoff score of 25.89 s [57]. The maximum duration of the test is 60-s [58]. The test will be carried out with 1 practice trial and 2 measured trials with a 60-s interval between each test. The longest time between the two tests will be recorded as the final score. The outcome will be discarded and replaced by a new test if: (1) participants move the tested foot; (2) the contralateral foot touches the floor.

Dynamic balance will be assessed using the Star Excursion Balance Test (SEBT), which is an effective means for determining reach deficits of CAI individuals [59]. There will be 8-direction including anterior, anteromedial, medial, posteromedial, posterior, posterolateral, lateral and anterolateral in the SEBT test. During the test, participants will be asked to stand at the center point using the involved limb and put their hands on their spina iliaca. Then, participants will be asked to use non-stance limb to reach in each direction as long as possible. In each direction, the farthest point of the great toe will be marked and recorded. The score will be discarded under the following conditions: (1) participants shift or lift the involved limb; (2) balance can not be maintained during the test. Before the official test, participants will have 2 practice trials to familiarize them with the test procedure and setting. To calculate the final SEBT score, the length of the lower limb measured from the anterior–superior iliac spine to the medial malleolus at the supine position will be used to divide the reach distance using the method described previously [60]. Besides, changes in SEBT scores will be examined to see if they reach the minimal detectable change in eight directions, including 6.73% (medial), 8.56 (anterior), 8.68 (anteromedial), 9.69 (anterolateral), 12.17 (lateral), 13.00 (posterior), 13.33 (posterolateral) and 13.36% (posteromedial) [61].

Proprioception will be evaluated by the Active Movement Extent Discrimination Apparatus (AMEDA). The AMEDA was designed to test the acuity of proprioception and it has been demonstrated to have good reliability and validity [62]. During the AMEDA test, one of 4 angles (10◦, 12◦, 14◦, and 16◦) of ankle inversion slope will be randomly generated for judgment. The Ankle Inversion Discrimination Apparatus for Landing (AIDAL), which has been determined to have higher ecological validity in assessing proprioception [63], will also be employed to assess ability to distinguish ankle inversion in landing tasks. Participants will be required to stand on the set-off platform with their eyes watching forward and then step off to land on a platform with one of four possible slopes (10◦, 12◦, 14◦, 16◦). Before the proprioception test, participants will be allowed to practice 4-time to enable them to remember the position sense associated with each slope. The formal session of tests, carried out by a blinded assessor using the random sequence, includes 40 trials (10 times for each angle at 10◦, 12◦, 14◦, 16◦) in AMEDA and AIDAL.

The isometric strength will be assessed using a hand-held dynamometer (precision 0.05%, input ± 15mv, measurement range -500 ~ 500N, Daysensor DY920, China), which has been tested as a reliable and valid tool in assessing isometric strength [64, 65]. It will be utilized to assess isometric strength in directions including inversion, eversion, dorsiflexion, and plantar flexion of the ankle joint. Each direction will be tested 2 times with a 60-s interval to obtain the average value for final analysis. Before strength measurement, all participants will be asked to be in a relaxed position. To ensure precision, the pelvis will be strapped onto the treatment bed.

In this study, functional performance will be estimated by the Side-Hop Test, described in another study [66]. All participants will be requested to hop 10 times (20 jumps together) across a 30 cm line as fast as possible. Before the formal test, they will be provided a chance to practice. This test will be conducted twice with a 3-min interval.

A handheld stopwatch will be used to record the time taken in the test. The faster result between the two formal tests will be selected as the final score. In addition, participants will be asked to report their degree of instability of the ankle joint (0–10 scale) during the test process.

### Brain plasticity

In the present study, transcranial magnetic stimulation (TMS) will be used to assess corticomotor alterations including cortical excitability, corticospinal inhibition, and representation of muscles. Single magnetic pulses (max 4 Tesla) will be delivered to the cortical representation of the lower leg muscles by a double-cone coil with TMS equipment (PowerMAG, Mag&More Inc., Germany). All participants will wear an elastic cap and sit on the chair with a relaxed lower limb. A wireless electrode of Delsy’s sEMG system (Trigno, Delsys Inc., Natick, MA, USA) will be placed on the muscle belly of the tibialis anterior and peroneus longus to collect the motor evoked threshold (MEP). The surface of these muscles will be shaved and cleaned with alcohol-impregnated tissues prior to electrode placement. The EMG signal will be collected at a sample rate of 2000 Hz and analyzed using the Matlab software (Matlab 2022a, MathWorks Inc., Natick, USA) with band-pass filters (high-pass of 10 Hz and low-pass of 500 Hz). Prior to the official test, the familiarization procedure will be conducted to reduce any apprehension caused by the magnetic stimuli. The method of identifying a hot spot will be in line with the one reported in a previous study [67]. The double-cone coil will be moved and delivers pulses (70% of magnitude, 5-s interval) to the vertex of the skull. The hot spot will be identified and marked until the maximum peak-to-peak MEPs (50 μV) are found. Subsequently, the stimulation magnitude on the hot spot decreases by 50% until MEP will not be elicited, and then increases by 1%. Rest motor threshold (RMT) will be defined as the stimulation magnitude by which 5 out of 10 pulses generate MEP > 20 μV [18]. Active motor threshold (AMT) will be defined as the least MEP intensity eliciting peak-to-peak MEP amplitude ≥ 100 μV in 5 out of 10 pulses. RMT and AMT will be presented as the percentage of maximum Tesla [68]. Corticospinal inhibition will be represented by the cortical silent period (CSP). According to previous studies, the CSP has been defined as the distance from the end of the MEP to the return of the mean EMG signal plus two times the standard deviation of the baseline EMG signal [69, 70]. The map area and the volume of cortical representation in the peroneus longus and anterior tibialis, which are used to represent an expansion of the cortical representation and the total excitability of cortical representation respectively, will be calculated by referring to the method reported previously [12]. All results in the TMS test will be collected in each group and used for statistical analysis.

Functional near-infrared spectroscopy (fNIRS) is a technique used to investigate patterns of cortical brain activation [71]. In this study, fNIRS will be implemented to investigate any alteration of functional plasticity after the WBV intervention. Oxyhemoglobin of the postcentral gyrus, the precentral gyrus, and the supplementary motor area will be recorded by the fNIRS system (NIRS Sport, NIRx Medical Technologies LLC, USA). In this system, there are 20 channels composed of 8 sources and 8 detectors. Two sorts of wavelengths, including 695 and 830 nm, and a frequency of 10 Hz will be adopted in the process of sampling data. The electrodes will be distributed to the targeted area according to the international 10/20 system. Positional information about each electrode will be obtained using a 3-dimensional digitizer (FASTRAK; Polhemus, Colchester, VT, USA) with 4 reference points (i.e., nasal root, parietal, left preauricular, and right preauricular) [13]. Before the formal test, participants will be given verbal instructions and a chance to familiarize themselves with the device. After this process, they will be asked to sit in the chair for 60 s to collect baseline cortical activity. Then, participants will stand with the involved leg on a firm surface and keep their hands on their hips. A sample lasting for 60 s will be conducted when standing with eyes opened and closed respectively. The interval between trials will be set at 60-s. Any recording during which participants lose their balance (e.g., removing hands from hips, shifting the feet, and touching the ground with the opposite limb) will be discarded, and retested until successful trials are obtained.

### Self-reported outcomes

In the current study, the CAIT and FAAM will be used to assess self-reported function. As a reliable questionnaire scale, the CAIT has been widely used to assess ankle instability for individuals with CAI [72]. Similarly, the Chinese version of the FAAM has also been tested and found to be valid [73]. These two measures will be performed using the paper version.

### Sample size

The needed sample size is calculated using the G-power software (version 3.1.9). The effect size is determined based on the value (0.35) previously reported in a study investigating cortical adaptations after balance training [40, 56]. Other parameters in the software of G-power are α error (0.05), power (0.8), number of groups (4), and number of measurements (3) [74]. Based on the above parameters, the total sample size given by the software is 64. Considering a 20% dropout rate, a total of 80 participants will be included, with 20 in each group.

### Statistics analysis

As all outcomes are continuous variables, they will be presented as mean ± standard deviation. The normality of variables will be tested by the Shapiro–Wilk test. All statistical analyses will be carried out using the SPSS software (Version 22.0, IBM Corp., Armonk, NY, USA) and *p* < 0.05 will be considered as a significant difference. Bonferroni adjustment will be performed in post-hoc analysis when statistical significance is found. A 1-way ANOVA analysis will be conducted to compare outcomes among the four groups. Pearson's correlation coefficient will be calculated to evaluate the relationship between alterations in sensorimotor function and plastic changes in the brain. The effect size (Cohen’s d, 95% confidence intervals) will be calculated to evaluate the magnitude of difference among groups. Ranges 0.2–0.49, 0.5–0.79, and > 0.8 are considered as small, medium, and large effect sizes respectively. A 2-way repeated-measure ANOVA analysis will be performed in terms of the different time points (i.e., baseline, post-test, and 2-week follow-up) of the various groups (i.e., WBVBT, BT, WBV, and control group). A correlation analysis will be conducted to determine the relationship between changes in sensorimotor deficits and neural plasticity. To estimate the efficacy and effectiveness of interventions, per‑protocol and intention-to-treat analysis will be conducted respectively if any participants withdraw.

### Monitoring

#### Safety and adverse effects

WBV may cause some potential side effects, including headache, dizziness, itch, erythema, and fatigue. However, these symptoms normally disappear soon after removing the vibration stimulus. Participants will be sent to a medical institution if their symptoms exacerbate. Before the commencement of assessment and treatment, researchers in the current study will complete the experimental checklist including examination of the physical and mental conditions of participants, the intervention equipment, and the operational circumstances, so as to guarantee the safety of the experimental process. In addition, adverse events in the whole process will be traced. Staff working in the ethical committee will check the recorded documentation to ensure that intervention and assessment will not produce physical or mental damage to participants.

#### Auditing

Demographic information (e.g., name, gender, etc.) will be collected in this research program. fNIRS data will be gathered in the electronic format. Data in paper format include signed informed consent, self-reported functions, and adverse events. The above data will be organized and stored in an encrypted computer. The data monitoring committee is made up of a staff working in the department of laboratory management and a senior professor who studies in the area of rehabilitation and physical education. These individuals are independent of researchers in the present study and any other potentially competing interests, and they will check the integrity of data in the trial process.

To improve compliance, participants will be told about the benefits of rehabilitative programs. Additionally, material rewards (e.g., gifts and cash) will be offered during the experimental process. One of the authors will document the information relating to the participation in intervention involving attendance, details of the training program, and participant feedback. Participants will be encouraged to report any difficulties and these will be recorded by the training supervisor. All data collected in the process of intervention will be transferred into an electronic version and stored on an encrypted computer managed by a professional staff member in the laboratory. In addition, these data will be checked once a week by the researcher manager.

#### Ethics and dissemination

The present study has been approved by the Medical Ethics Committee of the Department of Psychology and Behavioral Science of Zhejiang University (NO. 2,022,095). This ethical committee will be responsible for guaranteeing the rights, safety, and medical care of participants throughout the experiment. Further, the current study was registered in the Chinese Clinical Trial Registry (NO. ChiCTR2300068972) on 02 March 2023. The results of the current study will also be submitted to peer-reviewed journals and reported at conferences.

## Discussion

To the best of our knowledge, this will be a first study to assess the effect of WBV on brain plasticity in people with CAI. In addition, it will also investigate whether plastic changes in the brain correlate with the observed sensorimotor alterations. As consideration of plastic changes in the brain has become increasingly essential for understanding the mechanism of CAI, the current trial may provide evidence for the use of WBV in resolving sensorimotor deficits in patients with CAI.

Presumably because of the safety, efficacy, and efficiency, WBV has been the focus of increasing attention with respect to populations with frailty [75] and medical conditions [76, 77]. Our previous work, conducted by systematically reviewing the existing studies investigating WBV in CAI deficits, found that adding WBV to balance training could yield additional benefits in balance, strength, proprioception, and muscle activities of individuals with CAI [40]. Researchers involved in the previous studies speculated that the effect of WBV on the spinal reflex may be considered as one of the mechanisms, whereas the impact of WBV on the brain has been neglected to some extent [38, 53]. Since plastic changes in the brain were found to be closely associated with functional changes for individuals with CAI [14, 78], it is necessary to explore the role of brain plasticity in functional changes after intervention programs. On the other hand, it has been shown that plastic changes in the brain could be altered by the specific intervention forms, while the evidence related to WBV is lacking. For instance, 6-week stroboscopic balance training can change brain activities for athletes with CAI [21]. Similarly, transcutaneous electrical nerve stimulation and transcranial direct current stimulation were able to improve neural excitability [22, 79]. WBV has been demonstrated to be capable of modulating synaptic plasticity in animals [35, 36] and cortical activation among healthy people [33, 51], yet none of the studies have been done to examine whether WBV can induce neuroplastic changes in patients with CAI. Hence, it seems of value to investigate whether WBV can lead to benefits in sensorimotor function based on alterations occurring in the brain.

The stimulation of the motor cortex by TMS is a way to investigate the integrity of the brain in regards to muscle pathways and the functionality of cortical networks [80]. The changes from TMS (e.g., RMT, AMT, and CSP) observed in previous studies have indicated that there may be limitations in signal transmission from corticomotor systems to muscles around the ankle [12, 69, 81]. These alterations can be used to explain the changes in biomechanics [82] and kinematics [83] for individuals with CAI. In the current study, results of TMS assessments will be used to determine if there are associations between the sensorimotor changes caused by WBV intervention and alterations in the corticospinal pathway. fNIRS can be viewed as a non-invasive approach to assess functional activation of the brain through cerebral oxygenation changes evoked by haemodynamics [71]. A previous study using fNIRS to investigate alterations of cortical activation found significant differences in cortical-activation variability among individuals with CAI in comparison to a healthy control group [13], indicating that patients with CAI may have different patterns of cortical activation. In the present research, the changes in functional activations when participants stand in a one-legged stance can show how the cortical sensorimotor region responds to the instability of the ankle joints. In addition, the correlation between brain activation and alteration in sensorimotor function may provide evidence to support the strategy of taking brain activation as the target to achieve functional benefits.

Some limitations should be mentioned for the present study. First, compared to participants recruited at a university, patients from the community may have lower compliance because of their holiday, employment or family routines. Second, although participants will be requested to refrain from involvement in specific rehabilitation programs during the experiment, they will not be limited in regard to participation in regular exercises, which may be a factor influencing final outcomes. Third, despite the break provided between each of the tests, the results for brain plasticity and sensorimotor deficits may be interactively influenced, since these two measures are potentially associated [84]. Fourth, prognostic factors such as demographic features (e.g., age, sex), severity of lateral ankle sprain, anatomic configuration, functional performance, and initial treatment could potentially impact on the efficacy or mechanism of WBV, but this study may not be able to explain them clearly [85].

In conclusion, the main purpose of the current study is to explore the role of brain plasticity in the effects of WBV on sensorimotor deficits for individuals with CAI. The findings of this study will provide evidence for treating sensorimotor deficits from the perspective of brain plasticity. In addition, the results may also guide clinical practice when incorporating WBV into rehabilitation programs.

### Trial status

The current research protocol was approved on 26 December 2022 by the Medical Ethics Committee of the Department of Psychology and Behavioral Science of Zhejiang University. Then, one of the authors completed the registration procedure on 02 March 2023 on the official website named Chinese Clinical Trial Registry. The enrollment will be initiated on 01 May 2023 and ended up when the sample criteria are met.

## Data Availability

The datasets generated and/or analysed during the current study are not publicly available due to the privacy of the subjects, but are available from the corresponding author on reasonable request.

## Abbreviations

AIDAL: Ankle inversion discrimination apparatus for landing
AMEDA: Active movement extent discrimination apparatus
AMT: Active motor threshold
ANOVA: Analysis of variance
BT: Balance training
CAI: Chronic ankle instability
CAIT: Cumberland ankle instability tool
CONSORT: Consolidated standards of reporting trials
CSP: Cortical silent period
FAAM: Foot and ankle ability measure
fNIRS: Functional near-infrared spectroscopy
MEP: Motor evoked threshold
RMT: Rest motor threshold
SEBT: Star excursion balance test
SPIRIT: Standard protocol items recommendations for interventional trials
TIBT: Time-in-balance test
TMS: Transcranial magnetic stimulation
WBV: Whole-body vibration
WBVBT: Whole-body vibration and balance training
